# Does regional quota status affect the performance of undergraduate medical students in Japan? A 10-year analysis

**DOI:** 10.5116/ijme.6372.1fce

**Published:** 2022-11-30

**Authors:** Satoshi Ozeki, Sachiko Kasamo, Hiroyasu Inoue, Seiji Matsumoto

**Affiliations:** 1 Institutional Research Office, Asahikawa Medical University, Asahikawa, Japan

**Keywords:** Medical school admissions, academic performance, predictors of grade point average, regional quota, Japanese medical school

## Abstract

**Objectives:**

This study aims to determine whether there is a difference in the academic performance of medical students based on admission type and examine the extent to which entrance examinations predict their performance.

**Methods:**

This observational study utilized existing data from Asahikawa Medical University. Participants were 1057 medical students who had enrolled between 2010 and 2019. Analysis of variance and Tukey’s test were utilized to identify differences between admission types. The multiple linear regression explored predictors of cumulative grade point average for each type.

**Results:**

Analysis of variance showed significant differences in the National Center Test (F_(3, 1053)_ =70.78, p <0.001) and cumulative grade point average (F_(3, 1053)_ =3.93, p <0.01). Tukey’s post hoc test revealed that two types of general admission students (M=83.52, SD=3.22; M=85.57, SD=3.01) were significantly higher on the National Center Test than two types of regional quota students (M=81.61, SD=3.93; M=80.65, SD=3.61). The cumulative grade point average of a regional quota group (M=2.23, SD=0.34) was significantly higher than two types of general admissions (M=2.11, SD=0.36; M=2.12, SD=0.34). High school grade point averages and females were significant in predicting cumulative grade point averages for each admission (16.0–28.3% variance).

**Conclusions:**

Regional quota students earned a higher cumulative grade point average than those from general admissions, despite their significantly lower scores on the National Center Test. Enhanced utilization of regional quota admissions could become an effective strategy to increase the rural physician workforce.

## Introduction

Physician maldistribution, showing a relative shortage in rural areas, has become a common occurrence in many countries.^1^ The dearth of physicians can lead to significant health problems for those who reside in rural areas.^2^ The Japanese medical care system has also faced a relative shortage of physicians in rural areas for several decades.^3^^-^^5^ Internationally, countries such as the United States (U.S.) and Australia created admission policies to increase the number of future rural physicians. U.S. medical schools employ various approaches for recruiting and selecting prospective students who are likely to practice in a rural setting.^6^ In Australia, it was reported that the allocation of 25% of the admission capacity to students from rural backgrounds contributed to the expansion of the rural medical workforce.^7^ The Japanese Ministry of Health, Labour and Welfare introduced a similar regional quota admission system at Japanese medical schools, known as the regional frame, for which applicants who intend to practice medicine in designated regional areas are eligible to apply.^8^ In 2008, the Ministry permitted Japanese medical schools to increase their enrollment capacity for regional quota admissions, and many local universities incorporated it into their admission systems.^9^ The proportion of medical students entering through the regional quota admissions has increased from 5.2% in 2008 to 18.2% in 2020.^10^ The issue of physician maldistribution, however, has not been solved in Japan; the Ministry continues to explore more effective ways to utilize the regional quota admissions to solve this issue.^11^ With potential further use of the regional quota admissions in the future, it has become extremely important to ensure that the regional quota admissions are able to recruit prospectively high-performing medical students. Therefore, it is necessary to investigate whether the regional quota admissions screen medical school applicants in the same way as general admissions do.

In Japan, there is no entrance examination exclusively for medical school applicants – medical schools follow a standard Japanese university admission procedure. Most students enter medical schools directly from high school. Private universities have a certain degree of freedom on how to conduct their entrance examinations; however, national universities require applicants to take a standardized test: the National Center Test (NCT) for University Admissions. The NCT was developed to measure fundamental academic achievements at high school graduation level. It has five main subjects—Japanese; geography, history, and civics (GHC); mathematics; science; and foreign language—with various tests for each subject. Most national medical schools in Japan require applicants to take the NCT. General admissions usually comprise a two-stage process, where the NCT is utilized for screening purposes, followed by university-specific examinations, including advanced subject tests, interviews, and essays. Such university-specific subject tests, custom-created by each medical school, have been developed to ascertain the academic abilities of prospective students who are required to attain academic success at medical school.^12^ In contrast, the regional quota admissions commonly include an admission office examination (AO-EXAM: self-referral) and a recommendation examination (REC-EXAM: recommended by the high school). These admission types tend to place more emphasis on essays and interviews to determine students’ intention and motivation to work as physicians in selected regional areas. For this reason, there has been uncertainty among medical school educators about whether regional quota medical students, who are admitted without taking university-specific subject tests, can perform academically as well as their peers who had been admitted through general admissions.

With the recently expanded use of regional quota admissions, pathways to Japanese medical schools have become diverse, yet research on admission types and their connection to the academic success of medical students is very limited. A few investigations showed that medical students admitted under AO-EXAM or REC-EXAM received a higher grade point average (GPA) than those admitted through general admissions,^13^^-^^15^ although another study at a private medical school did not report a similar pattern.^16^ Regarding the predictors of academic success of medical students, previous studies did not reveal strong correlations between medical school GPA and the NCT^14^^,^^17^^,^^18^ or university-specific subject tests.^18^^,^^19^ Furthermore, research from a private university reported a weak correlation between entrance examinations and various tests during medical school.^20^ Several studies, conducted in Japan and internationally, suggested that academic performance at medical schools have comparatively high correlations with high school GPA^14^^,^^19^ and the female gender.^21^ Although past studies provide implications of the relationship between admission data and medical school performance, they are relatively outdated and do not reflect the recent expansion of the types of medical school admissions. To our knowledge, no published study centered its discussion on the regional quota admissions with respect to differences in medical school GPA and its predictors among various admission types. Given the potentially significant impact of the regional quota admissions on the future rural physician workforce in Japan, revealing the differences in academic performance of medical students by admission type can contribute to the advancement of medical school admissions for rural physicians. Admission processes containing the regional quota must be investigated to advance their effective usage. Therefore, this study intends to fill the knowledge gap in regional quota admissions of medical schools. Building on past literature, the primary purpose of this study is to determine whether the academic performance of medical students differs based on admission types and to explore its predictors by analyzing a decade’s worth of admission data. This study also aims to investigate the extent to which university-specific subject tests influence academic success during medical school. The three main research questions are as follows:

1. Do regional quota students, who are not assessed by university-specific subject tests, perform as well academically as those admitted under general admissions?

2. Among the variables available at the university entrance, which factors can predict medical school GPA?

3. Are university-specific subject tests a significant factor in predicting medical school GPA?

## Methods

### Study setting

This study was conducted at Asahikawa Medical University, a national medical university in Japan, established to improve medical services and welfare and to reduce disparities in medical care between urban and rural regions. To nurture individuals who can contribute to local medical care, the medical school of the university incorporated regional quota admissions into its admission system in 2008. Besides its openings for transfer students, the medical school offers two types of admissions: general admissions, and regional quota admissions. General admissions are further divided into first-round (Early-EXAM) and second-round (Later-EXAM) examinations, while regional quota admissions include AO-EXAM and REC-EXAM examinations. Applicants for the regional quota admissions come from areas close to the school who intend to receive post-graduate clinical training and practice medicine in designated local communities after graduation. All applicants must take the NCT. The required examinations and qualifications for each admission type are summarized in Table 1.

**Table 1:** Admission types and requirements at the study’s medical school

Admission		General admissions		Regional quota admissions	
Admission types		Early-EXAM^*^	Later-EXAM^†^	AO-EXAM^‡^	REC-EXAM^¶^
Recommendation		Not required		Self	High school
High school GPA		Not considered		Certain point required	
National Center Test		Cut-off score to proceed to university-specific examinations		Certain score required	
University-specific examinations	Subject tests	Mathematics, English	Science	Not required	
	Others	Group interview		Group interview, individual interview, essay	

Note: National Center Test includes 5 subjects: Japanese; Geography, history and civics; Mathematics; Science; Foreign language. *1st round general admission; †2nd round general admission; ‡admission office examination; ¶recommendation examination.

### Study design and participants

A retrospective observational study was adopted to answer the research questions. This study utilized existing data from Asahikawa Medical University. The study participants were medical students who had commenced their studies in the academic years of 2010 through 2019. Students who had withdrawn or were dismissed from the university, those with missing data on certain variables, and those admitted under the new admission type introduced in 2018, were excluded from this study. The total number of students for the study was 1057, the figures for each admission type were: 415 (296 male and 119 female) in Early-EXAM, 192 (139 male and 53 female) in Later-EXAM, 363 (201 male and 162 female) in AO-EXAM, and 87 (57 male and 30 female) in REC-EXAM.

This study was approved by the Asahikawa Medical University Research Ethics Committee. Since this study utilized existing data, written consent from individual participants was not required. The study information, including target participants and research purpose, were posted on the website of the Asahikawa Medical University Research Ethics Committee.

### Measures

#### National Center Test

The NCT is a standardized examination used to measure fundamental academic achievements at a high school graduate level.^22^ It was developed by the National Center for University Entrance Examinations, an independent administrative agency in Japan. It is held annually on two consecutive days at different locations in Japan. The NCT consisted of five subject areas, namely Japanese, mathematics, science, foreign language, and GHC. Each subject area was further divided into specific subjects. Each specific subject had four to six sections, with the total number of items ranging normally from 30 to 50 multiple-choice items. The purpose of the NCT was to improve the selection process of university admissions, and most universities have adopted it in Japan. Universities in Japan were permitted to utilize the results of the NCT in their own way by applying their own criteria and policies for student admissions. Thus, the total scores used in admission processes varied depending on the university, admission type, and year. Raw scores on each subject were converted into a percentage of 0–100% for data analysis.

#### University-specific examinations

University-specific examinations consisted of three subject tests (English, mathematics, and science), a group interview, an individual interview, and an essay. The subject tests had been developed by faculty members of the Entrance Examination Committee at Asahikawa Medical University to measure advanced academic skills and knowledge of the subjects that were not measured by the NCT. Unlike the NCT, those subject tests had fewer items, but with higher difficulty levels, the total number of items typically ranging from 10 to 20. They mostly consisted of items that required a short or essay-style answer. The essay and the group and individual interviews were intended to measure applicants’ motivation and aptitude for the medical profession, as well as basic general skills, such as communication and logical/critical thinking. The applicants had to respond to several questions in the interviews, during which their answers were scored by multiple assessors, with the average scores assigned as their overall scores. As described in Table 1, group interviews were required for all admission types. Subject tests for English and mathematics were required for Early EXAM, while science was compulsory for Later-EXAM. An individual interview and essay were mandatory for AO-EXAM and REC-EXAM. Because the maximum scores vary across different years and admission types, all the scores were converted into a percentage of 0–100% for data analysis.

#### High school GPA

Although different high schools may use different grading systems, the final high school GPA is generally reported on a 5-point scale system (1: very poor, 2: poor, 3: fair, 4: good, 5: excellent). A minimum high school GPA was a requisite for the AO-EXAM and REC-EXAM, while high school GPA in Early-EXAM and Later-EXAM was submitted but not considered for selection purposes. High school GPA was utilized for data analysis in all cases, regardless of its inclusion in the admission criteria.

#### University cumulative GPA

The Medical school assigned course grades, using a scale of 0 to 3 (0: fail, 1: pass, 2: good, 3: excellent). In the 2019 academic year, a letter grade of “outstanding” with a numerical value of 3.3 was introduced into the grading system. Since our system does not apply to students in 2019 only, this value was recalculated into 3 for purposes of the present study. The cumulative GPA indicates the mean of all the grades that students had earned through all the years they attended. Practice-oriented courses that assigned a pass or fail were not included in the calculation of the cumulative GPA.

### Data collection

Once this study had been approved by the Ethical Committee, we collected all the necessary data from the administrative offices of Asahikawa Medical University. Existing data on the entrance examinations were obtained through the Admission Division, while student university grades and high school GPA were obtained through the School Affairs Division.

### Data analysis

All data were analyzed using JMP 15 (SAS Institute Inc.). An analysis of variance (ANOVA) was performed to determine whether there was any statistical difference in the mean of the NCT and cumulative GPA among different admission types. When a statistical significance was observed, Tukey’s test was used to compare the different admission groups. Additionally, Pearson’s correlation coefficient between the cumulative GPA and each dependent variable was calculated, and a multiple linear regression analysis was conducted to explore predictors of cumulative GPA. The intercorrelations among predictor variables and variance inflation factors for each predictor variable were evaluated; the assumption of no multicollinearity was not violated. Due to the exploratory nature of this study, all the dependent variables were included in the multiple regression analysis. All statistical analyses were two-sided, at an alpha level of 0.05, to determine statistical significance.

## Results

The results of a one-way ANOVA identified a statistically significant difference in the means of all NCT subjects among the various admission groups (F_(__3, 1053)_ = 70.78, p < 0.001), as well as cumulative GPA (F_(3,1053)_ = 3.93, p < 0.01). The results of the post hoc comparisons using the Tukey tests are summarized in Table 2. As Table 2 indicates, the means of all NCT subjects in Early-EXAM (M = 83.52, SD = 3.22) and Later-EXAM (M = 85.57, SD = 3.01), were significantly higher than those of the AO-EXAM (M = 81.61, SD = 3.93) and REC-EXAM (M = 80.65, SD = 3.61). In addition, the cumulative GPA of REC-EXAM (M = 2.23, SD = 0.34) was significantly higher than Early-EXAM (M = 2.11, SD = 0.36) and Later-EXAM (M = 2.12, SD = 0.34).

Pearson’s correlation coefficients for all predictor variables against cumulative GPA by admission type were computed, as reflected in Table 3. Among all dependent variables within each admission type, high school GPA had the highest correlations, with cumulative GPA in Early-EXAM, r_(__413)_ = 0.33, p < 0.001, in Later-EXAM, r_(190)_ = 0.44, p < 0.001, and REC-EXAM, r_(85)_ = 0.45, p < 0.001, and the second highest in AO-EXAM, r_(362)_ = 0.27, p < 0.001. The female gender also showed relatively strong correlations with cumulative GPA within each admission type other than REC-EXAM, in Early-EXAM, r_(__413)_ = 0.21, p < 0.001, in Later-EXAM, r_(190)_ = 0.27, p = 0.001, and in AO-EXAM, r_(361)_ = 0.28, p < 0.001. Although not strong, GHC on the NCT recorded statistically significant correlations with cumulative GPA across all admission types. Mathematics, both in the NCT and university-specific subject tests, had statistically significant negative correlations with cumulative GPA in Early-EXAM. In the NCT, foreign language was identified as a statistically significant variable in Early-EXAM and AO-EXAM. Lastly, an individual interview was significantly correlated with cumulative GPA in AO-EXAM and REC-EXAM.

The results of the multiple regression analysis are presented in Table 4, with various statistically significant predictors of cumulative GPA by admission type. First, the model for Early-EXAM was statistically significant, F_(__10,404)_ = 10.26, p < 0.001, adj.R^2^ = 0.183. Female gender, high school GPA, GHC in the NCT, university-specific mathematics, and group interviews were statistically significant predictors of cumulative GPA in Early-EXAM. However, university-specific mathematics and group interviews influenced cumulative GPA negatively. Second, the multiple regression model of Later-EXAM statistically significantly predicated cumulative GPA, F_(__9,182)_ = 9.37, p < 0.001, adj. R2 = 0.283. Female gender and high school GPA, as well as GHC, science and foreign language in the NCT, were found to be statistically significant predictors of cumulative GPA in Later-EXAM although foreign language predicted cumulative GPA negatively. Third, the model for AO-EXAM was statistically significant, F_(__10,352)_ = 9.31, p < 0.001, adj. R^2^ = 0.187, with six statistically significant predictors: the female gender, high school GPA, GHC and science in the NCT, and group and individual interviews. Lastly, the regression model of REC-EXAM predicted cumulative GPA, F_(__10,76)_ = 2.64, p = 0.008, adj.R^2^ = 0.160, with high school GPA as the only statistically significant predictor in the model.

## Discussion

The results of our 10-year retrospective analysis based on four admission types show that medical students who were not assessed by university-specific subject tests under the regional quota admissions (AO-EXAM and REC-EXAM) obtained a higher cumulative GPA on average than those in general admissions (Early-EXAM and Later-EXAM).

**Table 2:** Tukey’s test comparisons of the means of the National Center Test and cumulative GPA by admission type

Variables		n	M	SD	Tukey’s test comparisons (p)		
					Early-EXAM^*^	Later-EXAM^†^	AO-EXAM^‡^
Five subjects in National Center Test							
Early-EXAM^*^		415	83.52	3.22	-	-	-
Later-EXAM^†^		192	85.57	3.01	< 0.001^***^	-	-
AO-EXAM^‡^		363	81.61	3.93	< 0.001^***^	< 0.001^***^	-
REC-EXAM^¶^		87	80.65	3.61	< 0.001^***^	< 0.001^***^	0.096
Cumulative GPA							
Early-EXAM^*^		415	2.11	0.36	-	-	-
Later-EXAM^†^		192	2.12	0.34	0.995	-	-
AO-EXAM^‡^		363	2.16	0.34	0.189	0.521	-
REC-EXAM^¶^		87	2.23	0.34	0.011^*^	0.041^*^	0.252

Note: The symbols ^*^, ^**^, and ^***^ denote p-values significant at < .05, < .01, and < .001, respectively.

^*^1st round general admission; ^†^2nd round general admission; ^‡^admission office examination; ^¶^recommendation examination.

**Table 3:** Pearson’s correlation coefficients (r) between cumulative GPA and predictors by admission type

Variables	Early-EXAM^*^ （n = 415）		Later-EXAM^†^ （n = 192）			AO-EXAM^‡^ （n = 363）		REC-EXAM^¶ ^（n = 87）		
	r	p	r	p		r	p	r		p
National Center Test										
Japanese	0.11	0.019^*^	0.03	0.690		0.10	0.068	-0.11		0.319
GHC	0.11	0.019^*^	0.21	0.003^**^		0.14	0.006^**^	0.24		0.024^*^
Mathematics	-0.10	0.043^*^	-0.10	0.178		-0.001	0.985	0.09		0.411
Science	-0.09	0.073	0.05	0.516		0.10	0.053	0.20		0.067
Foreign language	0.15	0.002^**^	0.04	0.602		0.12	0.019^*^	-0.04		0.742
University-specific examinations										
Mathematics	-0.10	0.045^*^	-	-		-	-	-		-
Science	-	-	0.02	0.752		-	-	-		-
English	0.07	0.151	-	-		-	-	-		-
Group interview	-0.09	0.078	-0.04	0.594		0.06	0.278	0.09		0.425
Individual interview	-	-	-	-		0.20	<0.001^***^	0.26		0.016^*^
Essay	-	-	-	-		0.07	0.205	0.02		0.856
High school GPA	0.33	<0.001^***^	0.44	<0.001^***^		0.27	<0.001^***^	0.45		<0.001^***^
Gender (Female)	0.21	<0.001^***^	0.27	<0.001^***^		0.28	<0.001^***^	0.02		0.838

Note: The symbols ^*^, ^**^, and ^***^ denote p-values significant at < .05, < .01, and < .001, respectively.

^*^1st round general admission; ^†^2nd round general admission; ^‡^admission office examination; ^¶^recommendation examination.

In particular, a statistically significant difference was identified between one of the regional quota admission, REC-EXAM, and general admission students. Furthermore, we discovered that the regional quota students scored statistically lower on the NCT than general admission students. Thus, we demonstrated that regional quota students were able to perform academically better than general admission students, although their academic skills were lower on the NCT, without being assessed by university-specific subject tests.

The current study’s findings are consistent with previous literature, indicating that AO-EXAM or REC-EXAM students tended to obtain a higher GPA than those obtained by general admissions.^13^^-^^15^ Those studies, however, had been conducted almost two decades ago, and analyzed only one of the two admission types. The importance of our study lies in the analysis of both AO-EXAM and REC-EXAM, reflecting the recent expansion of the Japanese medical school entrance examination system after the regional quota admissions had been introduced.

The multiple regression models identified in the present study show that admission data explained 16.0–28.3% of the variance in cumulative GPA (Table 4). These findings do not differ significantly from a previous examination, which reported an 11.7–25.1% variance in the average grades of medical students explained by admission data.^23^ High school GPA and female gender made comparatively large contributions in the prediction of cumulative GPA in our regression models, which is consistent with past investigations that suggested those variables as potential predictors of GPA.^14^^,^^19^^,^^24^ Among the variables utilized in our analysis, high school GPA was correlated significantly with cumulative GPA across all admission types. This suggests that students who possess strong fundamental academic skills, as demonstrated by a superior high school GPA, are likely to succeed academically at medical school. Hence, academic performance of medical students may be influenced by factors associated with high school GPA, such as effective study habits and test-taking strategies. As studies suggest the importance of investigating other elements, such as class attendance^16^ and strong motivation,^25^^,^^26^ it is imperative to examine factors that influence academic performance while studying at a medical school. Identifying such factors will yield valuable information on how to provide effective support to medical students with academic difficulties.

Japanese national medical schools’ general admissions typically select medical school applicants by university-specific subject tests created by each medical school. Such tests were created to assess advanced levels of English, mathematics, and science, which the NCT did not cover. The results of our study indicate that university-specific subject tests contributed little to the prediction of cumulative GPA. University-specific science and English were not significant predictors of cumulative GPA, while mathematics was identified as a significant predictor, yet its influence was small and negative. Therefore, we provide an important piece of empirical evidence that university-specific subject tests are not the sole influential factor when selecting students who are likely to succeed academically at medical schools.

Preliminary data suggest a higher pass rate for the Japanese National License Examination for Physicians by regional quota students.^27^^,^^28^ Since those students tend to practice in rural areas,^29^ the regional quota admissions are considered a promising measure to solve the issue of physician shortages in rural areas. As a regional medical university whose mission is to provide high-quality medical care to its surrounding areas, the regional quota admissions of the medical school examined by this study amount to half of its total enrollment capacity as of 2020. It is crucial to ensure that regional quota medical students perform academically as well as other admission types. Those living in rural areas should be able to access and receive the same high-quality medical care as those living in urban areas. As many countries face issues related to physician maldistribution^1^ our study could provide international implications for those countries that depend on or consider introducing a similar medical school admission strategy.

There are several limitations to our study. First, the present study discussed an advanced initiative of a regional medical school that made extensive use of the regional quota admissions to secure future rural physicians; the findings may, therefore, not be fully generalizable to other medical schools due to the diverse selection processes. In addition, our findings are limited to students at a single medical school. Second, university-specific examinations are not as reliable and valid as standardized examinations because they were individually created by each medical school. Although the university-specific examinations used in this study were carefully developed and examined by specialized faculty members in the Examination Committee at Asahikawa Medical University, they were internally created and assessed, but not tested nationally. Third, this study only analyzed those admitted to and who entered Asahikawa Medical University. It could not completely eliminate selection bias, which may influence the results. Notwithstanding these limitations, enhanced utilization of regional quota admissions, and ten years’ admission data used in the present study, can provide important pieces of evidence to support the implementation of an essential admission strategy for increasing the rural physician workforce.

## Conclusions

The results of the study affirm that regional quota students, despite their statistically lower scores on the NCT, and without their requirement to take university-specific subject entrance examinations, proved to perform academically as well as the general admission students. Particularly, the results of university-specific subject examinations contributed little to the prediction of the cumulative GPA.

**Table 4:** Regression models of the relationship between cumulative GPA and predictor variables by admission type

Variables	Early-EXAM^*^ （n = 415）			Later-EXAM^†^ （n = 192）			AO-EXAM^‡^ （n = 363）			REC-EXAM^¶^ （n = 87）		
	β	SE	p	β	SE	p	β	SE	p	β	SE	p
National Center Test												
Japanese	0.04	0.002	0.437	0.01	0.002	0.857	0.06	0.002	0.227	0.03	0.004	0.793
GHC	0.13	0.002	0.005^**^	0.19	0.003	0.003^**^	0.10	0.002	0.037^*^	0.16	0.004	0.146
Mathematics	-0.04	0.002	0.413	-0.08	0.003	0.252	0.03	0.002	0.546	0.03	0.004	0.795
Science	-0.08	0.003	0.096	0.14	0.004	0.043^*^	0.13	0.002	0.012^*^	0.10	0.005	0.407
Foreign language	0.09	0.003	0.052	-0.15	0.004	0.027^*^	0.07	0.003	0.165	-0.08	0.005	0.463
University-specific examinations												
Mathematics	-0.10	0.001	0.028^*^	-	-	-	-	-	-	-	-	-
Science	-	-	-	-0.08	0.002	0.221	-	-	-	-	-	-
English	0.01	0.001	0.758	-	-	-	-	-	-	-	-	-
Group interview	-0.15	0.001	0.002^**^	-0.11	0.002	0.106	0.10	0.001	0.036^*^	0.01	0.002	0.894
Individual interview	-	-	-	-	-	-	0.12	0.001	0.021^*^	0.07	0.003	0.562
Essay	-	-	-	-	-	-	0.04	0.001	0.417	0.03	0.002	0.739
High School GPA	0.30	0.033	<0.001^***^	0.44	0.046	<0.001^***^	0.22	0.064	<0.001^***^	0.39	0.186	0.001^**^
Gender (Female)	0.17	0.019	<0.001^***^	0.27	0.024	<0.001^***^	0.27	0.017	<0.001^***^	0.12	0.039	0.286
Adjusted R^2^		0.183			0.283			0.187			0.160	

Note: The symbols ^*^, ^**^, and ^*** ^denote p-values significant at < .05, < .01, and < .001, respectively.

^*^1st round general admission; ^†^2nd round general admission; ^‡^admission office examination; ^¶^recommendation examination; GHC, Geography, History, and Civics.

Providing an adequate number of physicians in rural areas has been a long-standing unsolved issue worldwide; hence, regional quota admissions may become a promising strategy to effectively select and secure future rural physicians who can achieve academic success at medical school. This study provides valuable insights for medical schools considering similar admission interventions to expand the rural medical workforce. Further research is required to examine additional factors of prospective students to predict student success and assess various learning outcomes of medical students apart from GPA. Additionally, future studies need to reveal motivations of medical students admitted through the regional quota admissions to work in the rural areas. Their opinions on medical schools’ curricula also need to be investigated to nurture physicians who can adequately contribute to rural medicine. This will enable medical curricula to be tailored to meet the specific needs of regional quota medical students.

### Acknowledgements

This study was supported by JSPS KAKENHI Grant Number JP20K18839. The authors would like to express their gratitude to Ms. Misako Sato for her assistance in data collection and the management for this study.

### Conflict of Interest

The authors have no conflicts of interest to declare.
